# Supplementary material for: Modeling glioblastoma heterogeneity as a dynamic network of cell states
Source: Mol Syst Biol. 2021 Sep 16;17(9):e10105. doi: 10.15252/msb.202010105 (PMC8444284; doi:10.15252/msb.202010105)
Supplement: Supplementary file 5 — Source Data for Figure 3 [file MSB-17-e10105-s001.zip › Figure3A_sourcedata/GSEA_3065/hallmarks_state2.GseaPreranked.1623416269049/HALLMARK_ESTROGEN_RESPONSE_LATE.html]

Details for gene set HALLMARK\_ESTROGEN\_RESPONSE\_LATE[GSEA]

|  || Dataset | state2 |
| Phenotype | NoPhenotypeAvailable |
| Upregulated in class | na\_pos |
| GeneSet | HALLMARK\_ESTROGEN\_RESPONSE\_LATE |
| Enrichment Score (ES) | 0.29935718 |
| Normalized Enrichment Score (NES) | 1.0111345 |
| Nominal p-value | 0.4474708 |
| FDR q-value | 0.4997411 |
| FWER p-Value | 1.0 |
Table: GSEA Results Summary

  

Fig 1: Enrichment plot: HALLMARK\_ESTROGEN\_RESPONSE\_LATE      
 Profile of the Running ES Score & Positions of GeneSet Members on the Rank Ordered List

  

| PROBE | GENE SYMBOL | GENE\_TITLE | RANK IN GENE LIST | RANK METRIC SCORE | RUNNING ES | CORE ENRICHMENT || 1 | TOP2A |  |  | 15 | 0.596 | 0.0505 | Yes |
| 2 | GINS2 |  |  | 37 | 0.506 | 0.0925 | Yes |
| 3 | CDC6 |  |  | 106 | 0.372 | 0.1178 | Yes |
| 4 | RBBP8 |  |  | 141 | 0.325 | 0.1425 | Yes |
| 5 | BTG3 |  |  | 173 | 0.295 | 0.1650 | Yes |
| 6 | CDC20 |  |  | 193 | 0.284 | 0.1879 | Yes |
| 7 | IDH2 |  |  | 218 | 0.272 | 0.2091 | Yes |
| 8 | NXT1 |  |  | 326 | 0.224 | 0.2173 | Yes |
| 9 | SNX10 |  |  | 400 | 0.200 | 0.2269 | Yes |
| 10 | IMPA2 |  |  | 564 | 0.169 | 0.2243 | Yes |
| 11 | HPRT1 |  |  | 573 | 0.168 | 0.2381 | Yes |
| 12 | PPIF |  |  | 580 | 0.166 | 0.2520 | Yes |
| 13 | CKB |  |  | 647 | 0.157 | 0.2586 | Yes |
| 14 | FABP5 |  |  | 796 | 0.138 | 0.2548 | Yes |
| 15 | EMP2 |  |  | 826 | 0.135 | 0.2636 | Yes |
| 16 | CCND1 |  |  | 840 | 0.134 | 0.2739 | Yes |
| 17 | PLK4 |  |  | 986 | 0.121 | 0.2689 | Yes |
| 18 | FKBP4 |  |  | 1079 | 0.113 | 0.2689 | Yes |
| 19 | KIF20A |  |  | 1094 | 0.112 | 0.2772 | Yes |
| 20 | MOCS2 |  |  | 1115 | 0.111 | 0.2847 | Yes |
| 21 | ARL3 |  |  | 1127 | 0.110 | 0.2932 | Yes |
| 22 | FKBP5 |  |  | 1180 | 0.106 | 0.2969 | Yes |
| 23 | OLFM1 |  |  | 1263 | 0.101 | 0.2969 | Yes |
| 24 | ETFB |  |  | 1334 | 0.096 | 0.2978 | Yes |
| 25 | FARP1 |  |  | 1441 | 0.090 | 0.2943 | Yes |
| 26 | DCXR |  |  | 1477 | 0.088 | 0.2982 | Yes |
| 27 | RNASEH2A |  |  | 1579 | 0.082 | 0.2946 | Yes |
| 28 | RABEP1 |  |  | 1602 | 0.082 | 0.2994 | Yes |
| 29 | STIL |  |  | 1784 | 0.073 | 0.2864 | No |
| 30 | PDLIM3 |  |  | 1841 | 0.071 | 0.2865 | No |
| 31 | PRKAR2B |  |  | 1863 | 0.070 | 0.2904 | No |
| 32 | RAB31 |  |  | 1977 | 0.065 | 0.2840 | No |
| 33 | CA2 |  |  | 2111 | 0.061 | 0.2751 | No |
| 34 | BAG1 |  |  | 2120 | 0.060 | 0.2795 | No |
| 35 | ASCL1 |  |  | 2318 | 0.053 | 0.2630 | No |
| 36 | ADD3 |  |  | 2401 | 0.051 | 0.2586 | No |
| 37 | XRCC3 |  |  | 2416 | 0.050 | 0.2616 | No |
| 38 | BCL2 |  |  | 2499 | 0.048 | 0.2569 | No |
| 39 | COX6C |  |  | 2515 | 0.047 | 0.2595 | No |
| 40 | SIAH2 |  |  | 2599 | 0.045 | 0.2545 | No |
| 41 | NAB2 |  |  | 2634 | 0.043 | 0.2546 | No |
| 42 | ITPK1 |  |  | 2684 | 0.042 | 0.2530 | No |
| 43 | IL17RB |  |  | 2712 | 0.041 | 0.2538 | No |
| 44 | FDFT1 |  |  | 2717 | 0.041 | 0.2569 | No |
| 45 | UGDH |  |  | 2897 | 0.036 | 0.2409 | No |
| 46 | SLC29A1 |  |  | 2930 | 0.036 | 0.2406 | No |
| 47 | SORD |  |  | 3056 | 0.033 | 0.2301 | No |
| 48 | TFPI2 |  |  | 3215 | 0.029 | 0.2157 | No |
| 49 | ISG20 |  |  | 3567 | 0.023 | 0.1800 | No |
| 50 | ACOX2 |  |  | 3627 | 0.022 | 0.1756 | No |
| 51 | SLC9A3R1 |  |  | 3823 | 0.018 | 0.1563 | No |
| 52 | GALE |  |  | 4116 | 0.014 | 0.1262 | No |
| 53 | GLA |  |  | 4388 | 0.009 | 0.0980 | No |
| 54 | PCP4 |  |  | 4628 | 0.005 | 0.0728 | No |
| 55 | NRIP1 |  |  | 4712 | 0.004 | 0.0642 | No |
| 56 | TST |  |  | 4725 | 0.004 | 0.0633 | No |
| 57 | JAK1 |  |  | 5116 | -0.002 | 0.0216 | No |
| 58 | RPS6KA2 |  |  | 5397 | -0.007 | -0.0078 | No |
| 59 | LARGE1 |  |  | 5611 | -0.010 | -0.0297 | No |
| 60 | METTL3 |  |  | 5770 | -0.013 | -0.0456 | No |
| 61 | DYNLT3 |  |  | 5958 | -0.016 | -0.0642 | No |
| 62 | HSPA4L |  |  | 6125 | -0.019 | -0.0804 | No |
| 63 | MAPT |  |  | 6242 | -0.021 | -0.0910 | No |
| 64 | DLG5 |  |  | 6469 | -0.025 | -0.1131 | No |
| 65 | UNC13B |  |  | 6545 | -0.026 | -0.1188 | No |
| 66 | SCARB1 |  |  | 6752 | -0.031 | -0.1382 | No |
| 67 | SEMA3B |  |  | 6759 | -0.031 | -0.1362 | No |
| 68 | AFF1 |  |  | 6774 | -0.031 | -0.1350 | No |
| 69 | MDK |  |  | 6783 | -0.031 | -0.1331 | No |
| 70 | XBP1 |  |  | 6931 | -0.035 | -0.1458 | No |
| 71 | TIAM1 |  |  | 6948 | -0.035 | -0.1445 | No |
| 72 | BLVRB |  |  | 7000 | -0.036 | -0.1467 | No |
| 73 | ELOVL5 |  |  | 7020 | -0.037 | -0.1455 | No |
| 74 | SLC2A8 |  |  | 7172 | -0.041 | -0.1581 | No |
| 75 | FOS |  |  | 7222 | -0.043 | -0.1596 | No |
| 76 | WFS1 |  |  | 7253 | -0.044 | -0.1590 | No |
| 77 | ABCA3 |  |  | 7275 | -0.044 | -0.1574 | No |
| 78 | SLC1A4 |  |  | 7280 | -0.044 | -0.1540 | No |
| 79 | FAM102A |  |  | 7294 | -0.045 | -0.1515 | No |
| 80 | NBL1 |  |  | 7312 | -0.045 | -0.1494 | No |
| 81 | PDCD4 |  |  | 7568 | -0.052 | -0.1721 | No |
| 82 | ALDH3A2 |  |  | 7763 | -0.059 | -0.1878 | No |
| 83 | TFAP2C |  |  | 7828 | -0.060 | -0.1894 | No |
| 84 | CA12 |  |  | 8005 | -0.067 | -0.2024 | No |
| 85 | DNAJC1 |  |  | 8012 | -0.068 | -0.1971 | No |
| 86 | NCOR2 |  |  | 8039 | -0.069 | -0.1939 | No |
| 87 | AMFR |  |  | 8048 | -0.069 | -0.1887 | No |
| 88 | SLC7A5 |  |  | 8067 | -0.070 | -0.1845 | No |
| 89 | TOB1 |  |  | 8169 | -0.076 | -0.1887 | No |
| 90 | CXCL12 |  |  | 8251 | -0.080 | -0.1904 | No |
| 91 | FLNB |  |  | 8416 | -0.090 | -0.2001 | No |
| 92 | MEST |  |  | 8534 | -0.099 | -0.2040 | No |
| 93 | TSPAN13 |  |  | 8713 | -0.112 | -0.2133 | No |
| 94 | FGFR3 |  |  | 8715 | -0.112 | -0.2036 | No |
| 95 | PLXNB1 |  |  | 8729 | -0.114 | -0.1951 | No |
| 96 | HOMER2 |  |  | 8730 | -0.114 | -0.1851 | No |
| 97 | ID2 |  |  | 8781 | -0.120 | -0.1800 | No |
| 98 | CPE |  |  | 8816 | -0.125 | -0.1727 | No |
| 99 | TPBG |  |  | 8829 | -0.127 | -0.1629 | No |
| 100 | PERP |  |  | 8836 | -0.127 | -0.1524 | No |
| 101 | MYOF |  |  | 8851 | -0.129 | -0.1426 | No |
| 102 | SLC16A1 |  |  | 8970 | -0.145 | -0.1426 | No |
| 103 | PRSS23 |  |  | 8975 | -0.145 | -0.1304 | No |
| 104 | SLC26A2 |  |  | 9005 | -0.149 | -0.1204 | No |
| 105 | DHCR7 |  |  | 9009 | -0.150 | -0.1076 | No |
| 106 | CELSR2 |  |  | 9020 | -0.152 | -0.0954 | No |
| 107 | CHPT1 |  |  | 9057 | -0.159 | -0.0853 | No |
| 108 | IL6ST |  |  | 9090 | -0.164 | -0.0744 | No |
| 109 | CAV1 |  |  | 9130 | -0.172 | -0.0635 | No |
| 110 | ABHD2 |  |  | 9250 | -0.213 | -0.0576 | No |
| 111 | ATP2B4 |  |  | 9314 | -0.243 | -0.0431 | No |
| 112 | CD9 |  |  | 9324 | -0.248 | -0.0224 | No |
| 113 | CD44 |  |  | 9407 | -0.397 | 0.0035 | No |
Table: GSEA details [plain text format]

  

Fig 2: HALLMARK\_ESTROGEN\_RESPONSE\_LATE: Random ES distribution      
 Gene set null distribution of ES for **HALLMARK\_ESTROGEN\_RESPONSE\_LATE**
